# Environmental and Serological Monitoring of Porcine Circovirus by Loop-Mediated Isothermal Amplification in Pig Farms

**DOI:** 10.3390/vetsci12101011

**Published:** 2025-10-18

**Authors:** Alexandre Lamas, Alejandro Garrido-Maestu, Gonzalo López-Lorenzo

**Affiliations:** 1 Food Hygiene, Inspection and Control Laboratory (Lhica), Department of Analytical Chemistry, Nutrition and Bromatology, Veterinary School, Campus Terra, Universidade de Santiago de Compostela, 27002 Lugo, Spain; alexandre.lamas@usc.es; 2 Laboratory of Microbiology and Technology of Marine Products (MicroTEC), Institute of Marine Research (IIM-CSIC), Eduardo Cabello, 6, 36208 Vigo, Spain; 3 Department of Animal Pathology (INVESAGA Group), Faculty of Veterinary Sciences, Universidade de Santiago de Compostela, Campus Terra, 27002 Lugo, Spain

**Keywords:** Porcine Circovirus type 2 (PCV2), environmental surveillance, loop-mediated isothermal amplification (LAMP), swine health monitoring

## Abstract

**Simple Summary:**

Porcine circovirus type 2 (PCV2) infections have a significant economic impact on pig production. Environmental and serological monitoring on farms is essential for controlling this viral disease. This study evaluated the use of a molecular technique called loop-mediated isothermal amplification (LAMP) for monitoring PCV2 in serum, air, and surface samples. This technique does not require specialized equipment and can be used on the farm itself, allowing it to be applied in monitoring programs without the need to send samples to a molecular biology laboratory.

**Abstract:**

Despite the widespread use of Porcine circovirus type 2 (PCV2) vaccination, subclinical infection persists and remains a concern due to its economic impact. Therefore, continuous herd-level monitoring is essential to assess the dynamics of this infection on farms and minimize its impact. This study evaluated the applicability of a loop-mediated isothermal amplification (LAMP) assay for PCV2 detection in serum, air, and surface samples collected under field conditions. In addition, a simplified Direct LAMP protocol, omitting DNA extraction, was compared with quantitative PCR (qPCR) as the reference method. A total of 360 samples from PCV2 vaccinated and unvaccinated fattening farms were analyzed. Diagnostic performance was assessed in terms of sensitivity, specificity, predictive values, and concordance with qPCR, using Cohen’s kappa coefficient (κ). LAMP showed higher agreement with qPCR (κ = 0.52) than Direct LAMP (κ = 0.16). Serum samples provided the most reliable results when DNA extraction was performed, reaching substantial agreement with qPCR (κ = 0.76). However, Direct LAMP applied directly to serum was negatively affected by inhibitory substances, resulting in a significant drop in sensitivity. In contrast both air and surface samples yielded comparable results between LAMP and Direct LAMP, without the need for DNA extraction. Notably, LAMP-based assays detected PCV2 circulation earlier than qPCR, particularly in environmental samples. These findings demonstrate the potential of LAMP as a practical alternative to qPCR for PCV2 monitoring. While DNA extraction remains essential for reliable detection in serum, Direct LAMP represents a promising strategy for environmental surveillance, enabling rapid, low-cost, and on-farm diagnostics.

## 1. Introduction

Porcine Circovirus Type 2 (PCV2) is the etiological agent of a considerable variety of syndromes that affect pigs including PCV2-systemic disease (PCV2-SD), PCV2-subclinical infection (PCV2-SI), PCV2-reproductive disease (PCV2-RD) and porcine dermatitis and nephropathy syndrome (PDNS) [1]. Nowadays, the clinical observation of these syndromes, mainly PCV2-SD, is limited due to the effectiveness and widespread use of PCV2 vaccination [2]. However, this infection remains a health concern in swine production due to its economic impact in PCV2-SI scenarios [3]. As a consequence, regular surveillance in swine farms is mandatory in order to guarantee the effectiveness of PCV2 vaccination or to characterize the epidemiological scenario in gilts or in outbreaks of other swine diseases [4,5,6,7].

In swine production, the development of innovative and effective diagnostic methods for disease surveillance has become a priority. In recent years, different methods have been proposed in order to obtain information regarding a pig population, to reduce the number of samples needed, and to optimize resources [8,9,10,11,12,13,14]. Samples like farm air, farm surfaces, oral and processing fluids, umbilical cords, and/ or skin wipes are interesting as they minimize the sampling time and the cost associated with surveillance. PCR, and specifically qPCR, is one of the most useful techniques for monitoring viruses at herd level [15]. This molecular technique stands out for its speed and specificity, but has the disadvantage of requiring specific equipment, dedicated facilities, and qualified personnel. Therefore, exploring the use of PCR alternative techniques is a required field of research as it could reduce the laboratory procedures and facilitate the detection of infections in surveillance protocols.

In the year 2000 Notomi et al. published the principle of loop-mediated isothermal amplification, LAMP [16]. LAMP is an isothermal technique in which amplification takes place at a constant temperature (60–70 °C). Therefore, it does not require specific equipment, and when this technique is combined with visual detection methods, amplification can be performed in a thermoblock or water bath, which are standard pieces of laboratory equipment. Ever since, its applicability has increased exponentially due to its suitability for decentralized setup analysis, low infrastructure requirements and ease of performance, among other advantages over other nucleic acid amplification techniques [17]. In recent years, various LAMP methods have been developed for virus detection as enterovirus, Hepatitis A or Norovirus [18,19,20,21]. One of the limitations common to all molecular techniques is that they require a preliminary step of DNA/RNA isolation. This not only increases response time but also increases the cost associated with the analysis. The DNA/RNA isolation step is usually necessary both for the lysis of the cells/viruses where the genetic material is found and for its purification. The latter is often of great importance in techniques such as qPCR, as samples may contain different substances that inhibit the reaction. However, LAMP is usually more resistant to the presence of inhibitory substances, making it an interesting option for use in samples without prior DNA extraction [22,23,24].

The objective of the present work was to assess the suitability of a LAMP-based assay for the specific detection of PCV2 in clinical, environmental and surface samples to trace infection in vaccinated and unvaccinated fattening farms with and without DNA isolation.

## 2. Materials and Methods

### 2.1. Sample Collection and Farm Description

A stock of 360 samples from a previous study was used [8]. From these samples, 250 corresponded to serum, 60 to air and 50 to different farm surfaces. The sampling protocol for each type of sample is described in Table 1.

These samples were taken in two commercial fattening farms with different health status against PCV2 infection: Farm A housed 364 unvaccinated pigs; and Farm B housed 381 PCV2 vaccinated pigs with a commercial vaccine at 4 weeks of age. Both farms have the same structure (central alley with pens to each side, housing approximately 15 pigs in each one totally slated floor, a totally solid wall, one hopper and one nipple drinker), ventilation (automated lateral windows depending on the indoor temperature) and farm management (all-in/all-out with cleaning and disinfection between batches and supplied with nine-week-old piglets). Both farms were visited when the pigs were 10, 12, 14, 16 and 18 weeks old, analyzing from each visit 25 serum samples, 6 air samples and 5 surface samples.

### 2.2. Sample Laboratory Pretreatment

Serum samples were pooled (five samples/pool), including only pigs from the same farm and visit. Serum pools were kept at −30 °C until posterior procedures.

Filters from each air sample were dissolved adding 5 mL of sterile phosphate-buffered saline with 0.05% Tween 20 (PBST). This solution was homogenized for one minute and left to settle down for 15 min. After that, 1 mL of supernatant were transferred to a sterile Eppendorf tube and kept at −30 °C until further procedures.

To each tube with, surface swabs of 5 mL of PBST were added. Then, they were vortexed for 1 min and subsequently left to settle down for 15 min. After that, 1 mL of supernatant was transferred to a sterile Eppendorf tube and kept at −30 °C until further procedures.

### 2.3. LAMP Primer Design

**Figure 1:**
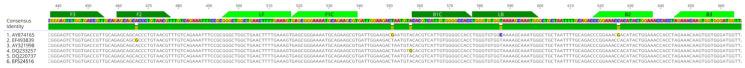
Clustal W PCV2 alignment of sequences AY874165, AY321998, EF493839, EF524516, DQ233257 and DQ220737. On top of the consensus sequence, the relative position of the newly designed LAMP primers, is provided. Figure generated with Geneious Prime 2023.2.1 (Biomatters Ltd., Auckland, New Zealand).

A novel LAMP assay was designed. To this end, six PCV2 genomes were retrieved from NCBI (https://www.ncbi.nlm.nih.gov/), namely AY874165, AY321998, EF493839, EF524516, DQ233257 and DQ220737. The genomes were aligned with Clustal W installed in Geneious (Geneious Prime^®^ software Version 2024.0.7 (Biomatters Ltd., Auckland, New Zealand) and rep gene was selected for primers design. The consensus sequence obtained was used for primer design taking advantage of the freely available, online software Primer Explorer V5 (https://primerexplorer.eiken.co.jp/e/). A suitable set of regular and loop primers was selected, avoiding mismatches in the 5′ and 3′ ends of the oligonucleotides. The sequences are provided in Table 2, and their relative positions within the amplified fragment are shown in Figure 1. A 350 bp fragment of the rep gene in which the designed primers hybridize was inserted into a plasmid pMA-RQ (AmpR) (Invitrogen, ThermoFisher Scientific, Waltham, MA, USA) and used as positive control.

### 2.4. Laboratory Analysis

Each sample was analysis using three different procedures.

#### 2.4.1. Procedure A: qPCR Assay (DNA Extraction + PCV2 qPCR)

DNA extraction was carried out in all samples (serum pools, air samples and surface samples) from 200 µL of sample using a commercial DNA extraction kit (High Pure PCR Template Preparation Kit, Roche Diagnostics GmbH, Mannheim, Germany) following the manufacturer’s instructions. The obtained DNA was collected in 100 μL of elution buffer and kept at −30 °C until qPCR analysis. In addition, an exogenous internal control (EXOone EXIC, EXOPOL S.L., Zaragoza, Spain) was added to each air and surface sample to identify possible qPCR inhibition.

Air and surface samples negative to PCV2 qPCR analysis were re-extracted with a second commercial DNA extraction kit (Nucleospin^®^ Soil, Macherey-Nagel GmbH & Co KG, Düren, Germany) following the manufacturer’s instructions, using 200 µL of starting material and collecting the extracted DNA in 100 μL of elution buffer. The same exogenous internal control was also added to each sample.

qPCR analysis was performed using a commercial kit that targets the ORF2 gene (EXOone PCV2 oneMIX, EXOPOL S.L., Zaragoza, Spain), following the manufacturer’s instructions. qPCR-positive and -negative controls were supplied by the manufacturer and were used in each run. A sample was considered positive when Cq ≤ 40 for the PCV2 detection channel. All qPCR reactions were run in duplicate on an Applied Biosystems ABI Prism 7500 thermocycler (Thermo Fisher Scientific, Waltham, MA, USA).

#### 2.4.2. Procedure B: LAMP Assay (DNA Extraction + PCV2 LAMP)

DNA extraction was carried out in all samples (serum pools, air samples and surface samples) from 200 µL of sample using the High Pure PCR Template Preparation Kit as described in Procedure A. The reactions were performed in a final volume of 20 µL, composed of 2 µL of template DNA, 12 µL of Fast Master Mix (ISO-004, OptiGene, Horsham, UK), 1 µL of 20X primer mix (final concentrations detailed in Table 2), 0.4 μL of CXR Reference Dye (final concentration 50 nM, Promega, Madison, WI, USA), and the remaining volume was filled with nuclease-free water. All experiments were performed in a QuantStudio™ 12 k Flex Real-Time PCR system (Applied Biosystems, Thermo Fisher Scientific, Waltham, MA, USA) set at 65 °C for 30 min with fluorescence acquisition every 30 s. The amplification was followed by melt-curve analysis consisting of heating at 95 °C for 15 s, cooling to 80 °C for 20 s, and heating up to 95 °C in 0.05 °C/s increments with fluorescence acquisition after each increment. Samples showing both technical replicates with amplification times below 20 min, whose Tm values matched those of the positive control ±2 SD, were considered positive.

#### 2.4.3. Procedure C: Direct PCV2 LAMP Assay

DNA extraction was not performed in any samples. The PCV2 LAMP was applied directly to the samples following its laboratory pretreatment. The setup of the experiments was the same as detailed above in Section 2.4.2. However, the criteria for positive samples when performing the Direct LAMP assay were the same as those with DNA extraction but the amplification time was increased up to 25 min; in this sense, samples with times higher than 25 were considered negative.

### 2.5. Statistical Analysis

The results obtained were analyzed in terms of positive and negative samples reported for each technique (qPCR, LAMP, and Direct LAMP) and for each sample type (serum, air, and surface). Concordance, as a measure of the level of agreement between LAMP and Direct LAMP results compared with the reference method (qPCR), was assessed using the standardized Cohen’s kappa coefficient (κ) [25]. This index was applied both to evaluate the overall concordance and to assess agreement within each sample type.

## 3. Results

**Figure 2:**
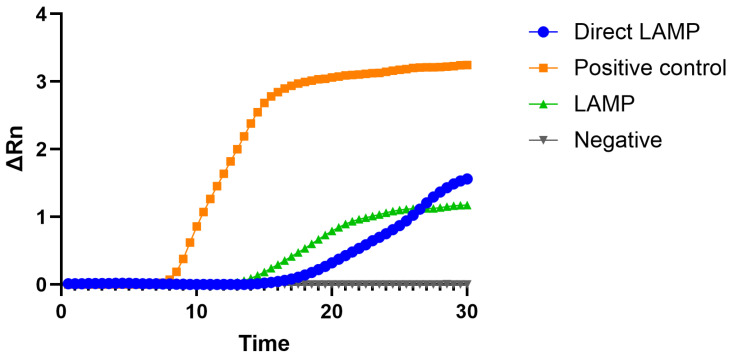
Typical amplification plots obtained with the LAMP assays with when adding purified DNA or when applying the Direct LAMP approach in an air sample.

Differences were observed in LAMP with and without prior DNA extraction. In Figure 2 a typical result depicting these differences can be observed.

### 3.1. Comparison Among PCV2 qPCR, PCV2 LAMP and Direct PCV2 LAMP

**Figure 3:**
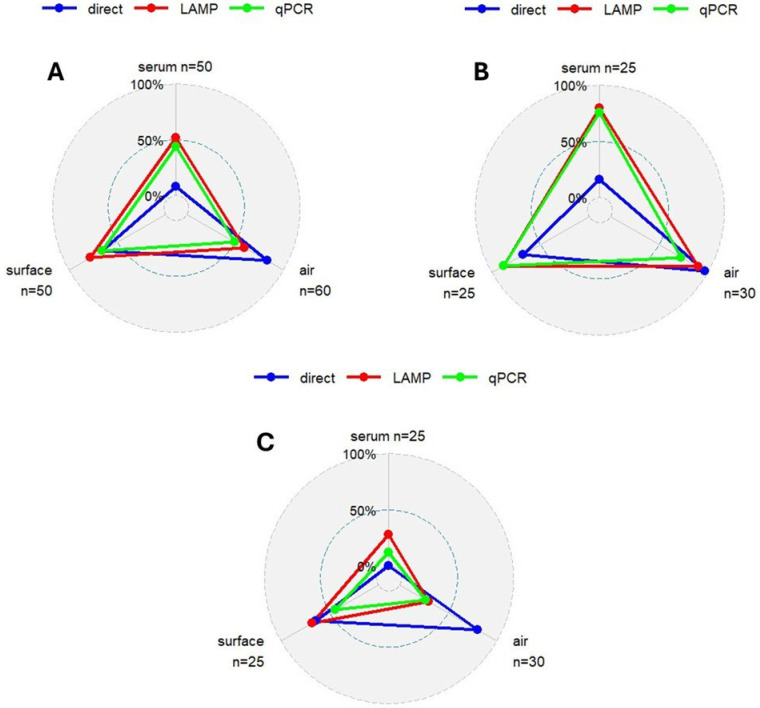
(**A**) Percentage of positive samples with the three different detection techniques used. (**B**) Percentage of positive samples with the three different detection techniques in the three sample types in Farm A (PCV2 unvaccinated pigs). (**C**) Percentage of positive samples with the three different detection techniques in the three sample types in Farm B (PCV2 vaccinated pigs).

Table 3 and Figure 3 show all the results obtained for each technique and each type of sample. The LAMP assay yielded the highest number of positive samples, followed by Direct LAMP, while the reference method, qPCR, detected the lowest number of positive samples.

Total concordance was higher between qPCR and LAMP (76.25%) than between qPCR and Direct LAMP (58.12%). This trend was also reflected in Cohen’s Kappa values, with a moderate agreement observed between qPCR and LAMP (κ = 0.52), and only slight agreement between qPCR and Direct LAMP (κ = 0.16). Similarly, sensitivity, specificity, positive predictive value (PPV), and negative predictive value (NPV) were all higher for LAMP compared to Direct LAMP when evaluated against qPCR. These results are summarized in Table 4.

Regarding the samples analyzed, noteworthy differences were observed. Overall, the best performance metrics were obtained with serum samples, followed by air samples, and finally surface samples. However, specific observations can be made for each sample type. First, serum samples showed substantial agreement between LAMP and qPCR (κ = 0.76), with all other evaluated parameters exceeding 80%. In contrast, when Direct LAMP was applied, these values declined markedly, particularly with a drastic reduction in sensitivity. Second, air samples had the lowest positive predictive value (PPV) for both LAMP and Direct LAMP compared to qPCR, and Direct LAMP showed the lowest specificity. Third, surface samples analyzed with LAMP showed a lower agreement with qPCR as, even though its SE was slightly higher than that of air samples, the SP was lower (similar observation for PPV and NPV, respectively). Similarly, when these samples were analyzed by Direct LAMP, the degree of agreement with qPCR significantly lower given the fact that even though its SP was slightly higher, the SE was significantly lower, this being more clearly reflected in the PPV and NPV, where the first was slightly higher than in air samples; however the NPV was drastically lower. It is important to note that the observed lower performance of LAMP in this type of sample may be motivated by their lower number. A more detailed breakdown of the performance metrics by sample type is provided in Table 4.

Regarding the samples that tested positive when using the reference method (qPCR), it is noteworthy that all samples with a Cq value below 30 were also positive when using the LAMP assay. However, this was not the case with Direct LAMP, in which several of these samples, particularly serum samples, tested negative. At higher Cq values, the inconsistency in positive detection increased for both LAMP and Direct LAMP when compared to qPCR. Interestingly, some of the samples that tested negative in qPCR were found to be positive in the other assays. Among serum samples, 5 out of 28 qPCR-negative samples were positive when using LAMP, while all remained negative with Direct LAMP. For air samples, of the 30 qPCR-negative samples, 12 were positive in LAMP and 24 in Direct LAMP. Lastly, for surface samples, out of 17 qPCR-negative samples, 10 and 12 were positive in LAMP and Direct LAMP, respectively.

### 3.2. PCV2 Health Situation

#### 3.2.1. Unvaccinated Pigs (Farm A)

In Farm A, a total of 69 samples tested positive and 11 negative in LAMP, corresponding to 86% and 14%, respectively. Similar results were obtained with qPCR, with 63 samples testing positive (79%) and 17 negative (21%). When analyzed by Direct LAMP, 50 samples were positive (62.5%) and 30 were negative (37.5%).

Regarding the type of sample, clear differences were observed among the three different detection approaches. Using serum samples, 80%, 76% and 16% of samples tested positive in LAMP, qPCR, and Direct LAMP, respectively. When the analyses were performed on air samples, the percentage of positive results was 90% for LAMP, 73% for qPCR, and 97% for Direct LAMP. Lastly, when analyzing surface samples, 88% tested positive in both LAMP and qPCR, while 68% were positive in Direct LAMP. These results are graphically depicted in Figure 3.

#### 3.2.2. Vaccinated Pigs (Farm B)

In Farm B, a total of 33 samples tested positive and 47 tested negative in LAMP, representing 41% and 59%, respectively. When analyzed by qPCR, 22 samples were positive (28%) and 58 tested negative (72%). Direct LAMP detected 40 positive samples, corresponding to 50%.

Regarding the type of sample, differences were also observed among the different procedures. The percentage of positive serum samples was 28%, 12%, and 0% for LAMP, qPCR, and Direct LAMP, respectively. Regarding air samples, 30%, 27%, and 80% tested positive in LAMP, qPCR, and Direct LAMP, respectively. Finally, for surface samples, the corresponding positive rates were 68%, 44%, and 64%. These results are illustrated in Figure 3.

### 3.3. Monitoring PCV2 Infection

#### 3.3.1. Unvaccinated Pigs (Farm A)

**Figure 4:**
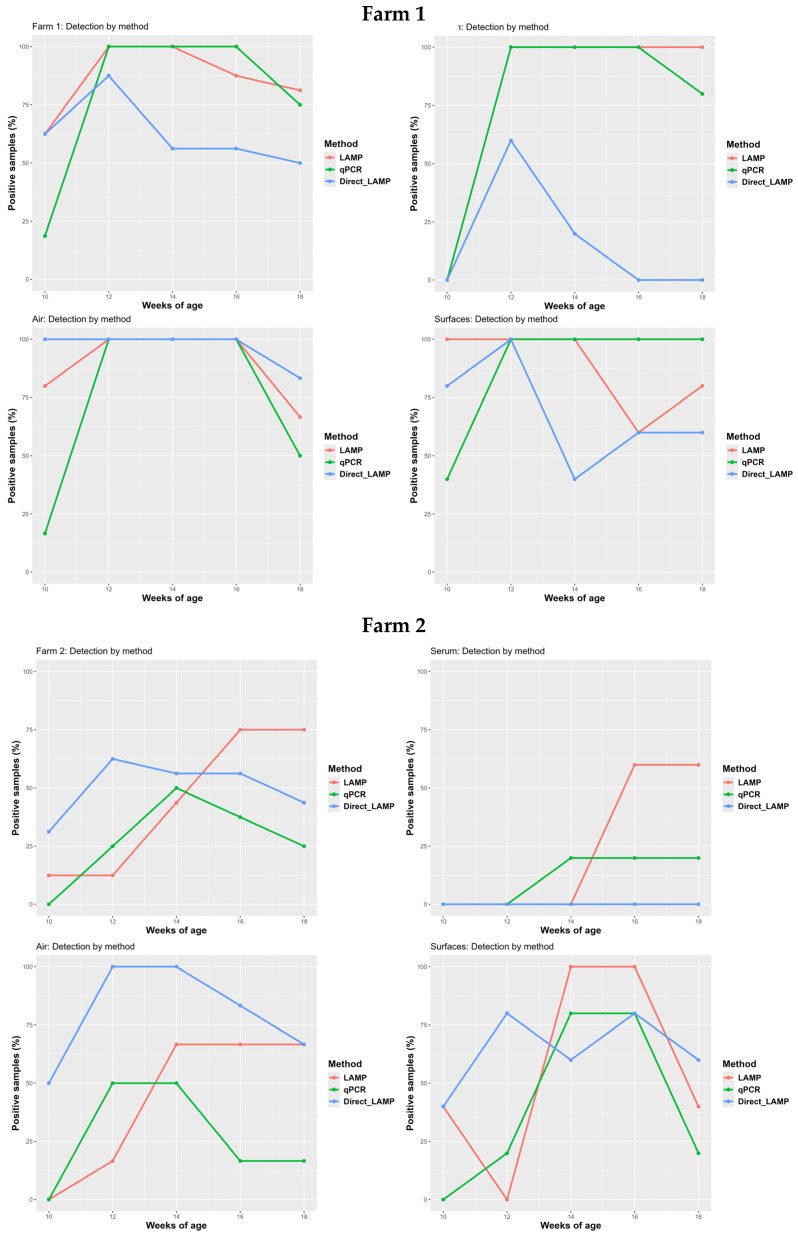
Evolution of the number of positive samples by method and farm according to the sampling weeks.

At 10 weeks of age, the presence of PCV2 was detected by all diagnostic procedures. Of the 16 samples collected, 10 tested positive in both LAMP and Direct LAMP, while only 3 were positive in qPCR. In the subsequent visit, the number of positive samples increased through all methods. From 14 weeks of age onward, the percentage of positive samples gradually declined for all diagnostic assays. This trend is consistent with the expected dynamics of PCV2 infection, as confirmed by qPCR monitoring of serum samples. Notably, the decline in the percentage of positive samples was more pronounced with Direct LAMP, primarily due to a marked reduction in the number of positive results from serum and surface samples (Figure 4).

#### 3.3.2. Vaccinated Pigs (Farm B)

At 10 weeks of age, PCV2 infection was already detectable on the farm; however, it was identified exclusively through LAMP and Direct LAMP assays in air and surface samples. In subsequent visits, the number of positive samples increased across all diagnostic methods (qPCR, LAMP, and Direct LAMP), reaching peak detection at 12, 14, or 16 weeks of age, depending on the assay used (Figure 4).

Under a PCV2 vaccination scenario, it is particularly noteworthy that all serum samples analyzed by Direct LAMP consistently tested negative throughout the study. Additionally, while the percentage of positive samples detected by qPCR either peaked or remained stable over time, LAMP consistently yielded higher positivity rates, particularly in serum and air samples. In contrast, the positivity rates for surface samples were more comparable between LAMP and qPCR.

## 4. Discussion

As far as we know, this is the first study to apply loop-mediated isothermal amplification (LAMP)-based diagnostics for the detection of a virus, Porcine Circovirus Type 2 (PCV2), in air and surface samples collected under field conditions. While PCV2 detection by LAMP has been previously reported in serum and tissue samples with successful results [28], the current findings expand its applicability to environmental matrices. These results highlight the potential of LAMP as a sensitive tool not only for individual animal diagnosis but also for the environmental surveillance of viral pathogens, in agreement with what Spiteri et al. reported in 2024 [29]. Furthermore, they emphasize the importance of considering several factors when applying LAMP directly to samples of different nature, as matrix composition, viral load, and sample processing requirements may significantly influence assay performance.

Our results are consistent with those reported by Rajkhowa et al., Spiteri et al., and Roumani et al., showing comparable values for sensitivity, specificity, concordance and k value between qPCR and LAMP [28,30,31]. In the specific case of PCV2 serum samples, the slightly lower values obtained in our study across these parameters compared to Rajkhowa et al. may be attributed to differences in the primer sets used for LAMP, as well as to variations in viral load among serum samples [28]. In this regard, a low number of qPCR-positive samples with Cq values above 30 tested negative in LAMP. This observation aligns with previous findings suggesting that certain LAMP primer sets may have limitations in detecting low concentrations of PCV2, particularly in samples with high Cq values [32,33].

The present study demonstrates that, when implementing LAMP-based procedures under field conditions, several factors must be considered both the sample type and the specific LAMP protocol used. Serum samples exhibited high reliability when the LAMP was performed following a DNA extraction, yielding results comparable to those obtained by qPCR. However, when LAMP was applied directly to serum without prior nucleic acid extraction, the number of concordant positive samples was drastically reduced. This may be attributed to the presence of inhibitory substances such as proteins, hemoglobin, lipids, immunoglobulins, or enzymes commonly found in serum, which can interfere with DNA polymerase activity [34,35]. These inhibitors are typically removed during DNA extraction, which likely explains the contrasting results observed between LAMP and Direct LAMP in serum samples. The lack of an Internal Amplification Control, capable of tracing this phenomenon in LAMP reactions, as previously reported [36], is a limitation which should be addressed in future works.

In contrast, the potential presence of inhibitors does not appear to significantly affect air and surface when LAMP is applied directly in these types of samples, note that increasing the number of samples withing these types will strengthen this observation. This is particularly important as it suggests that Direct LAMP may be reliably used for non-invasive environmental monitoring without the need for prior nucleic acid extraction, thereby simplifying the workflow and opening the possibility of performing this diagnosis directly on-farm. However, caution must be taken when applying this approach as indicated by several of the performance parameters obtained in this particular type of samples, such as the PPV in the range of 50–60%, or the NPV ~30%, as detailed in Table 4. Additional work to further confirm our results would be of great interest. Consequently, this represents one of the principal contributions of our study, highlighting the feasibility of implementing Direct LAMP for the field detection of viral pathogens in farm environments. Considering this, the validation of colorimetric Direct LAMP in environmental samples seems a promising direction for future studies.

Regarding both PCV2 health statuses, the three diagnostic procedures yielded broadly comparable results, with the exception of Direct LAMP applied to serum samples in the farm with unvaccinated pigs and to air samples in the farm with vaccinated animals. Overall, a higher proportion of positive samples was observed across all sample types and diagnostic methods in the farm with unvaccinated animals, which is consistent with the higher prevalence of infection confirmed by the analysis of serum samples, and with the higher PCV2 shedding in the absence of vaccination [37,38,39]. The reduction in PCV2 infection levels and viral spread within the herd can be detected by all three diagnostic techniques, making LAMP procedures suitable tools for evaluating vaccine efficacy in terms of reducing PCV2 shedding.

Focusing on PCV2 infection monitoring, both LAMP and Direct LAMP detected PCV2 circulation in both farms as early as the first sampling visit. In contrast, qPCR only identified positive samples in the farm with unvaccinated pigs at that time. This earlier detection by LAMP and Direct LAMP compared to qPCR was exclusively attributable to the analysis of air and surface samples, as illustrated in Figure 4. This is a noteworthy finding, suggesting that LAMP may offer advantages for early detection of infections when using environmental samples. In fact, this observation is supported by Zhang et al., who reported that the detection limit of LAMP is lower than that of qPCR when applied to air samples under experimental conditions; however, as indicated previously, addition work to further support our observations would be of great interest [40]. Moreover, this could explain why, at least in the farm with vaccinated animals, the proportion of positive samples detected by LAMP and Direct LAMP was higher in air and surface samples, a trend that became more pronounced toward the end of the monitoring period. This elevated detection rate in environmental samples during the later stages might be attributed not only to active viral shedding by infected pigs but also to the resuspension of PCV2-contaminated dust particles previously deposited on surfaces by infected animals [41].

## 5. Conclusions

This study demonstrates the potential of LAMP and Direct LAMP as effective diagnostic tools for monitoring PCV2 infection under field conditions, particularly when applied to environmental samples. Both methods successfully detected PCV2 circulation earlier than qPCR, with positive results in air and surface samples during the initial sampling, even in vaccinated herds.

The reliability of LAMP regarding qPCR was strongly influenced by the type of sample and the sample preparation protocol. While LAMP performed on extracted DNA from serum showed high concordance with qPCR, Direct LAMP applied to serum showed a marked reduction in diagnostic performance. In contrast, environmental samples such as air and surface samples, did not exhibit such limitations, and Direct LAMP remained effective even without prior DNA extraction, thus being a rapid and cost-effective tool. This finding is particularly relevant for the implementation of on-farm diagnostics, supporting the feasibility of rapid and simplified pathogen detection directly in the field.

## Figures and Tables

**Table 1:** Protocol to take serum, air and surface samples.

Sample	Procedure
Serum	Venipuncture in jugular vein
Air	Using the MD8 Airport (Sartorius AG, Göttingen, Germany) with sterile gelatine filters of 80 mm in diameter and a pore size of 3 μm (Sartorius Stedim Biotech GmbH, Göttingen, Germany): a flow of 50 L air for 30 min for each air sample.
Surfaces	Using sterile cotton swabs: - Central alley: 100 steps were taken wearing polyethylene boot covers. Afterwards, the bottom of both boot covers were swabbed in zigzag. - Pen railing: 1 m of length was swabbed in zigzag. - Hopper: eight different hoppers, sampling in each one an area of 25 × 25 cm. - Pen wall: eight different pens, sampling in each one an area of 25 × 25 cm. - Pen floor: the same protocol described in central alley.

**Table 2:** Set of LAMP primers.

Primer	Sequence (5′ → 3′)	Final Concentration (nM)
PCV2_F3	GGG AGT CTG GTG ACC GTT	200
PCV2_B3	CCA TCC CAC CAC TTG TTT CT	200
PCV2_FIP	ACG CTT CTG CAT TTT CCC GCT C *tttt* AGC AGC ACC CTG TAA CGT	800
PCV2_BIP	CAC GTC ATT GTG GGG CCA CC *tttt* TTC CAG TAT GTG GTT TCC GG	800
PCV2_LF	ACT TTC AAA AGT TCA GCC AGC CC	400
PCV2_LB	TGG GTG TGG TAA AAG CAA ATG G	400

In FIP and BIP the “*tttt*” sequence represents a polyT linker.

**Table 3:** Positive and negative samples for each analytical method.

Samples		qPCR No. (%)	LAMP No. (%)	Direct LAMP No. (%)
Positive		85/160 (53.12%)	101/160 (63.12%)	90/160 (56.25%)
	Serum	22/50 (44.00%)	26/50 (52.00%)	4/50 (8.00%)
	Air	30/60 (50.00%)	36/60 (60.00%)	53/60 (83.33%)
	Surfaces	33/50 (66.00%)	39/50 (78.00%)	33/50 (66.00%)

**Table 4:** Concordance and Cohen’s Kappa (k) measure of agreement.

Compared Analytical Methods			Positive Concordant Samples (True Positives) No.	Negative Concordant Samples (True Negatives) No.	Total Concordance No. (%)	κ Value	SE (%)	SP (%)	PPV (%)	NPV (%)
qPCR vs. LAMP	Total		74	48	122 (76.25%)	0.52	87.06%	64.00%	73.27%	81.36%
		Serum	21	23	44 (88.00%)	0.76	95.45%	82.14%	80.77%	95.83%
		Air	24	18	42 (70.00%)	0.40	80.00%	60.00%	66.67%	75.00%
		Surfaces	29	7	36 (72.00%)	0.32	87.88%	41.18%	74.36%	63.64%
qPCR vs. Direct LAMP	Total		54	39	93 (58.12%)	0.16	64.29%	51.32%	59.34%	56.52%
		Serum	4	28	32 (64.00%)	0.2	18.18%	100%	100%	60.87%
		Air	29	6	35 (58.33%)	0.17	96.67%	20.00%	54.72%	85.71%
		Surfaces	21	5	26 (52.00%)	−0.07	65.63%	27.78%	61.76%	31.25%

Calculation of the SE, SP, PPV, NPV and κ was performed as previously described [26,27].

## Data Availability

The original contributions presented in this study are included in the article. Further inquiries can be directed to the corresponding author(s).
